# A transformational moment for London Journal of Primary Care

**DOI:** 10.1080/17571472.2018.1486501

**Published:** 2018-07-30

**Authors:** Mayur Lakhani, David Morris

**Affiliations:** a Royal College of General Practitioners. 30 Euston Square, London, UK; b Centre for Citizenship and Community, School of Social Work, Care and Community, University of Central Lancashire, Preston, UK

This bumper issue of London Journal of Primary Care (LJPC) provides a fitting end to its ten years of activity. It signals a PAUSE to prepare for a new stage of translating into practice the ideas that LJPC has been promoting about community-oriented integrated care.

LJPC started in 2008 as the Journal of the three London RCGP faculties. Now it has out-stripped its original intention. It has gone beyond London RCGP Faculties and is now an international, peer-reviewed journal registered on PubMed Central. It has become more than journal – it is a *community of practice* that includes policy-makers, practitioners, managers, educationalists and researchers who want to understand community-oriented integrated care. It is concerned with more than primary care – it is concerned with integrating the contributions to health and care of everyone in society.

The ideas developed by LJPC in the past ten years will be built on in the future by the University of Central Lancashire, with RCGP collaboration. This is likely to include a network of case study sites, a new publishing platform, and a new name to enable further progress in understanding how to build and sustain collaboration at local level for health and for care.

This final issue contains papers that demonstrate the power of the LJPC approach.

• LJPC has emphasised co-production of papers to address complex, ‘wicked’ problems, like improving health and treating diseases at the same time. For example LJPC facilitated the co-production of a paper that proposed a set of actions that primary care can do to improve mental health [1], and this became adopted nationally by the RCGP. The paper in this issue, ‘*Evaluating case studies of community oriented integrated care’*, is another example of co-production. It has 29 authors and summarises learning of LJPC members about how a publishing platform can develop a community of practice. They reached their conclusions through a series of conferences, workshops and email discussions.
• LJPC has emphasised the need to develop collaborating sites to observe what happens over time in complex changing situations [2]. The Liverpool Open Eye photographic gallery is one such collaborating site; in this issue they provide a powerful case study of the potential of art (in this case socially-engaged photography) to improve health and reveal health need. LJPC has published papers on the power of Art before, notably Sue Hallam’s review of the power of music to enhance wellbeing in elderly people [3].
• LJPC has nurtured many papers into publication that publicise good grass-roots work. The paper by Hassanally on homeless mortality in this issue is another example of good grass-roots work done by GPs with disadvantaged people whose stories are often not told.
• LJPC has developed ways for members to learn from and with each other. One method has been to publish papers in advance of a conference to enhance learning at the conference (e.g. papers to support discussions about community-oriented integrated care at the London City Health conferences and to support ethics workshops at the annual RCGP conferences). This issue includes seven papers written by conference delegates in preparation for the (June 2018) international conference on child mental health in London. Authors from Malaysia, Japan, Hong Kong and Oman describe health need and innovations in their countries.
• LJPC has influenced policy, both locally and nationally. Miller and Breakwell’s literature review in this issue describes the factors that influence a family to agree to organ donation. This is another example of a paper that intends to influence policy by clarifying the most significant issues.
• LJPC values diverse insights. In this issue, insights from other countries are to be found in Halpern & Berney’s account of rural Tanzania, and in Yaman’s study of exercise prescription in Turkey.
• This stage of LJPC fittingly ends with a paper by Francesco Carelli about poetry, art and parenting, inspired by an exhibition in Milan of the paintings of Marc Chagall. Francesco wrote in the very first LJPC issue in June 2008 and many times since. His mingling of ideas from art and health are inspiring – **What colour do you feel today?**

We want to congratulate the LJPC editorial team for having maintained a consistently high level of quality in the 380 papers it has published over the past ten years and very particularly, we want to thank and congratulate Paul Thomas as Editor in Chief for his tireless leadership of both the editorial team and the journal itself since its inception. Paul’s commitment to the ideas of the journal and to the innovation in primary and community oriented care that they embody has been, and will continue to be a source of immeasurable inspiration to those who will now ‘take over the baton’ and lead the next stage of this vital agenda. To those, we also offer our very good wishes.

Mayur Lakhani *Professor, President of Royal College of General Practitioners, 30 Euston Square, London, NW1 2FB*
Mayur.Lakhani@rcgp.org.uk
David Morris *Professor, Mental Health, Inclusion and Community, Director, Centre for Citizenship and Community, School of Social Work, Care and Community, University of Central Lancashire, Preston PR1 2HE*
dmorris1@uclan.ac.uk

